# Feasibility analysis of carbon nanofiber synthesis and morphology control using a LPG premixed flame

**DOI:** 10.3762/bjnano.16.45

**Published:** 2025-04-23

**Authors:** Iftikhar Rahman Bishal, Muhammad Hilmi Ibrahim, Norikhwan Hamzah, Mohd Zamri Mohd Yusop, Faizuan Bin Abdullah, I Putu Tedy Indrayana, Mohd Fairus Mohd Yasin

**Affiliations:** 1 Department of Thermo-Fluids, Faculty of Mechanical Engineering, Universiti Teknologi Malaysia, 81310 Johor Bahru, Malaysia; 2 High Speed Reacting Flow Laboratory (HiREF), Universiti Teknologi Malaysia, 81310 Johor Bahru, Malaysia; 3 Advanced Membrane Technology Research Center, Universiti Teknologi Malaysia, 81310 Johor Bahru, Malaysia; 4 Department of Chemistry, Faculty of Science, Universiti Teknologi Malaysia 81310 Johor Bahru, Malaysia; 5 Department of Physics, Udayana University 80231 Badung, Bali, Indonesia

**Keywords:** carbon nanofiber (CNF), equivalence ratio, flame synthesis, liquefied petroleum gas (LPG), nanomaterial synthesis

## Abstract

Flame synthesis using liquefied petroleum gas (LPG) as the precursor gas to produce carbon nanofibers (CNFs) is an economical alternative to conventional chemical vapor deposition methods using single-component fuels such as methane and ethylene. Though LPG is a commercially viable source for carbon-based nanomaterials, the understanding of the effects of a LPG flame on CNF growth is very limited. Therefore, the present study is to analyze the feasibility of CNF growth in a premixed LPG flame using a one-dimensional flame at varying equivalence ratios. The effects of flame equivalence ratio on the CNF morphology and crystallinity are then analyzed systematically. In the present study, a diffusion flame was used to check the stability of the flame at different flow rates, followed by establishing a premixed flat flame of LPG. An optimum height above burner of 10 mm at which the temperature is around 650 °C was used in the synthesis process. Zirconia beads impregnated with nickel nitrate catalyst have been employed. Dense CNF growth with an average diameter of 77.9 nm was observed at an equivalence ratio of 1.8; as the equivalence ratio was reduced to 1.6, the average diameter of CNF increased by 46% to 114 nm, with amorphous carbon observed. The said observation is due to the effects of the increased flame temperature as the equivalence ratio approaches stoichiometry conditions from the rich side. This increases the nucleation rate, which in turn increases the catalyst particle size and the amount of free carbon atoms, producing CNFs with larger diameters and amorphous carbon. According to Raman analysis, the grown CNFs have a high number of defects, which may be good for applications where defective nanomaterials are desirable to improve the component performance. The work has proven that flame synthesis of CNFs using commercial LPG is feasible, paving the way for further exploration into cost-efficient CNF production with potential industrial applications.

## Introduction

Carbon nanotubes (CNTs) and carbon nanofibers (CNFs) have gained significant interest because of their distinctive properties and their wide range of applications in nanotechnology [1–3]. CNTs are a modified version of CNFs, with the primary distinction between CNTs and CNFs being the arrangement of graphene layers. CNFs have a cylindrical or conical structure, with their diameter varying from a few to several hundred nanometers [4]. In CNFs, carbon atoms form covalent bonds, resulting in a three-dimensional hexagonal graphene sheath. These nanofibers can have three different structural configurations including herringbone, tubular, and platelet configurations [4–5]. A premixed flame of liquefied petroleum gas (LPG) can be used as a fuel source for carbon nanomaterial growth processes. A premixed flame is a specific combustion mode where the fuel and oxidizer are thoroughly mixed before ignition. LPG is a cheap industrial material used as a carbon source to produce carbon nanomaterials [6]. The application of CNFs includes, but is not limited to, energy storage in batteries and supercapacitors, electronics, drug delivery, tissue engineering, and implants [7].

Methods of CNT/CNF synthesis include (a) chemical vapor deposition, (b) arc discharge, (c) flame synthesis, and (d) laser ablation [1]. Flame-assisted synthesis is a promising method for efficient and continuous one-step production. Various flame configurations, fuel types, and catalytic materials can be employed to produce desired growth and morphology. Flames potentially enable the synthesis of carbon nanomaterials in large quantities at significantly lower cost than that of other methods currently available [8].

In some applications, CNFs can be of great importance. Defective CNFs are utilized to build composites with specific thermal conductivities as part of thermal insulation materials. They can also be used to make materials that combine strength and flexibility, for example, in electronics or damping devices. The presence of defects in CNFs also contributes to the percolation threshold, which is a fundamental parameter controlling electrical conductivity and crucial for certain applications such as sensors, where accurate modulation is required [9].

An initial work by Naha et al. developed a model to enhance the understanding and optimization of CNT/CNF synthesis in flame environments. An ethylene/air co-flow, non-premixed flame was used with a catalyst substrate of iron, nickel, and platinum wires of 0.1–0.25 mm diameter. The study found that carbon monoxide is a major contributor to CNT formation in flames, and the model also showed that flame synthesis can be faster and yield higher throughput compared to some recent works. The CNT/CNF growth rates decrease with increasing height above the burner because of lower temperatures and reduced CO concentrations [10].

A custom-made chamber device for flame fragments deposition with three stainless steel inlet tubes for LPG, oxygen, and nitrogen was used to synthesize CNTs. TEM images revealed a 0.35 nm interplanar spacing, showing high crystallinity and a thin amorphous layer [11]. In a separate study, CNFs were synthesized using acetylene and plasma-enhanced chemical vapor deposition with nickel as the catalyst and hydrogen as the plasma source. SEM and TEM characterization showed vertically aligned CNFs. This method highlights the impact of plasma conditions and gas ratios on CNF morphology and alignment [12]. An experiment by Li et al. showed CNT growth at decomposition temperatures of 550–650 °C [13].

Also, CNFs were produced by loading a stainless-steel autoclave with 7.50 mL of diethyl ether and a solution containing 1.00 g of zinc powder and 0.50 g of iron powder. The sealed autoclave was heated to 650 °C and kept overnight for the hydrothermal reaction. After cooling to room temperature, the product was purified with 1% HCl solution, distilled water, and ethanol, then vacuum-dried at 50 °C for about 4 h. The final product was nearly pure CNFs, as shown by FESEM images. TEM images indicated an average CNF diameter of 100 nm. Raman spectra showed a strong, narrow peak at 1607 cm^−1^ and a few broad peaks, indicating less graphitization [14]. In another work, a high-density inductively coupled plasma chemical vapor deposition method yielded vertically aligned CNFs using acetylene and hydrogen on a p-type Si wafer with a 10 nm Ni catalyst layer at 20 mTorr and 550 °C. CNF length increases with deposition time, but density decreases because of the detachment of smaller CNFs [15].

A study by Ibrahim et al. showed that CNT growth extends from near the flame sheet toward the centerline within the fuel stream, where carbon sources are abundant. Growth regions cease where temperatures drop below the minimum required for growth [16]. The study highlights that high temperatures and a rich supply of carbon are critical for sustaining CNF growth. A study by Kumar et al. also suggests the importance of maintaining an optimal carbon concentration for efficient SWCNT growth [17].

A study by Zhuang et al. used electrospinning to synthesize CNFs. Polyacrylonitrile was used a carbon precursor and phosphomolybdic acid was added to improve the structure and the conductivity of CNFs. A voltage of 15 kV was applied to a stainless steel needle containing the solution and an aluminum foil covered drum was placed 18 cm from the needle to collect the nanofibers. The flow rate of the solution was 0.5 mL/h. The as-prepared nanofibers underwent thermal treatments, and it was found that the nanofibers exhibited optimal properties after treatment at 850 °C. The SEM and TEM image revealed nanofiber morphology, and Raman spectroscopy showed the characteristic *I*_G_ and *I*_D_ bands of CNFs [18].

Catalytic chemical vapor deposition was conducted by Hammel et al. to synthesize CNFs using a tube furnace. The experiment used a nickel-based catalyst and diluted acetylene as the source of carbon. The temperature was kept below 700 °C, and the efficiency of conversion of carbon precursors into fibers found was 70%. The diameter found was approximately 100 nm, and the TEM and SEM characterization revealed the morphology and the internal structures of the fibers [19].

Ruiz-Cornejo et al. discussed several applications of CNFs. Thin CNFs have a large surface area and are used for adsorptive hydrogen storage. Also, CNFs are used as electrode materials in supercapacitors. They can also be used for water purification and carbon capture and storage [20].

LPG gas contains a flammable mixture of hydrocarbon gases, mainly propane, butane, isobutane, propylene, and butylene. The lower flammability limit (LFL) is 1.81%, and the upper flammability limit (UFL) is 8.86% for upward propagation of the flame. Whereas, for downward propagation of the ﬂame, the LFL and UFL are 1.87% and 7.69% of LPG, respectively. The lean flammability limit for upward propagation occurs at an equivalence ratio, Φ, of 0.53, equivalent to 1.81% LPG by volume. Conversely, the rich flammability limit is observed at an equivalence ratio of 2.80, corresponding to 8.86% LPG [21].

A study showed that the Raman peaks of CNFs are 1350 cm^−1^ for *I*_D_ and 1592 cm^−1^ for *I*_G_. Generally, the formation of sp^2^-hybridized carbon atoms is often correlated to Raman spectra having G peaks at 1550–1600 cm^−1^, indicating the crystallinity. Similarly, a D peak at 1250–1450 cm^−1^ often correlates to defects and disorders of the sp^2^-hybridized sidewalls, while the G′ peak at 2500–2900 cm^−1^ represents photon–phonon interactions [22].

To the best of our knowledge, the present study describes the first flame synthesis using a LPG premixed flame and a spherical substrate for CNF growth. The characteristics of the LPG premixed flame are studied with an initial diffusion flame to compare the stability to achieve a premixed flame. Flames with different equivalence ratios were systematically used to confirm the feasibility of the synthesis process and the effects of flame conditions on the morphology of the grown CNF.

## Experimental

### Preparation of impregnated beads

Zirconia beads of 0.30 mm diameter were selected as a substrate. The beads were cleaned by sonication in ethanol followed by rinsing with distilled water; the rinsed beads were dried in an oven to remove contaminants. The cleaned zirconia beads were impregnated with nickel catalyst to be used as a substrate for the synthesis process. In this process, 20.30 g of nickel nitrate hexahydrate were mixed with 70 mL of deionized water using a magnetic stirrer to prepare a 1 M nickel nitrate solution. The solution of nickel nitrate was used to impregnate the pores of the spherical zirconia beads. After saturation, the impregnated zirconia beads were dried at a temperature of 90 °C. Finally, a reduction process was carried out in a furnace at a temperature of 500 °C to transform the nickel nitrate into nickel oxide on the zirconia substrate.

#### Experimental setup

**Figure 1:**
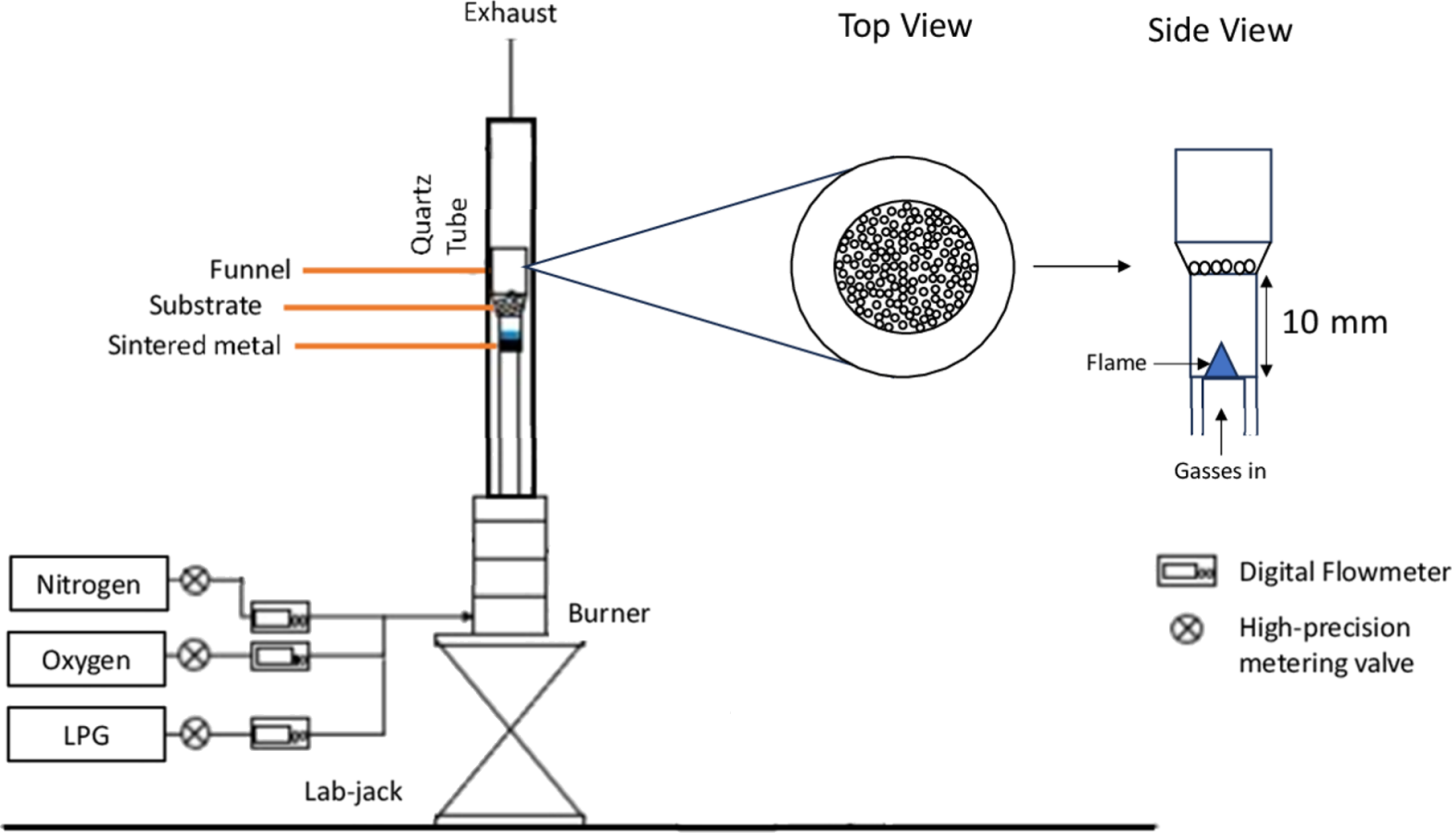
Schematic of the setup.

The setup of the premixed flame burner with a sintered metal outlet is shown in Figure 1. The burner comprises a premix chamber to ensure the thorough mixing of the gasses and a concentric nozzle tube outlet with diameters of 17 mm. A quartz tube with an outer diameter of 50 mm is placed around the burner nozzle to ensure that the flame is not hindered by atmospheric air. During CNF synthesis, a funnel with height above burner (HAB) of 10 mm was used to hold the substrate. The LPG and oxidizer gasses were fed through one inlet of the burner. A digital flowmeter was used to control the flow of gasses, and the burner was placed on a stand. The ideal combustion of the premixed flame was achieved by mixing LPG, oxygen, and nitrogen at 0.18, 0.65, and 1.90 slpm, respectively. The mixture produced a premixed flat flame with a secondary diffusion flame at an equivalence ratio of 1.80.

#### Flame and CNF characterization

The premixed LPG flame was measured using a type-K thermocouple with an accuracy of ±2.2 °C. The bead size of the thermocouple was 1 mm in diameter. The thermocouple was attached to a traversing system that can be used to move the thermocouple bead to specific spots in the flame. To ensure high-precision placement of the thermocouple within the flame, a two-axis positioning system was employed with an Arduino-based control unit. The positioning system has 1 mm accuracy with *x*- and *y*-axis traverse ranges of 100 and 200 mm, respectively.

The grown CNFs were characterized by field-emission scanning electron microscopy (FESEM, Zeiss Crossbeam 340) for morphological analysis. Raman spectroscopy (HORIBA XploRA PLUS, 532 nm) was carried out to analyze the signature spectra of the grown CNFs.

## Results and Discussion

### Flame characterization and temperature

**Figure 2:**
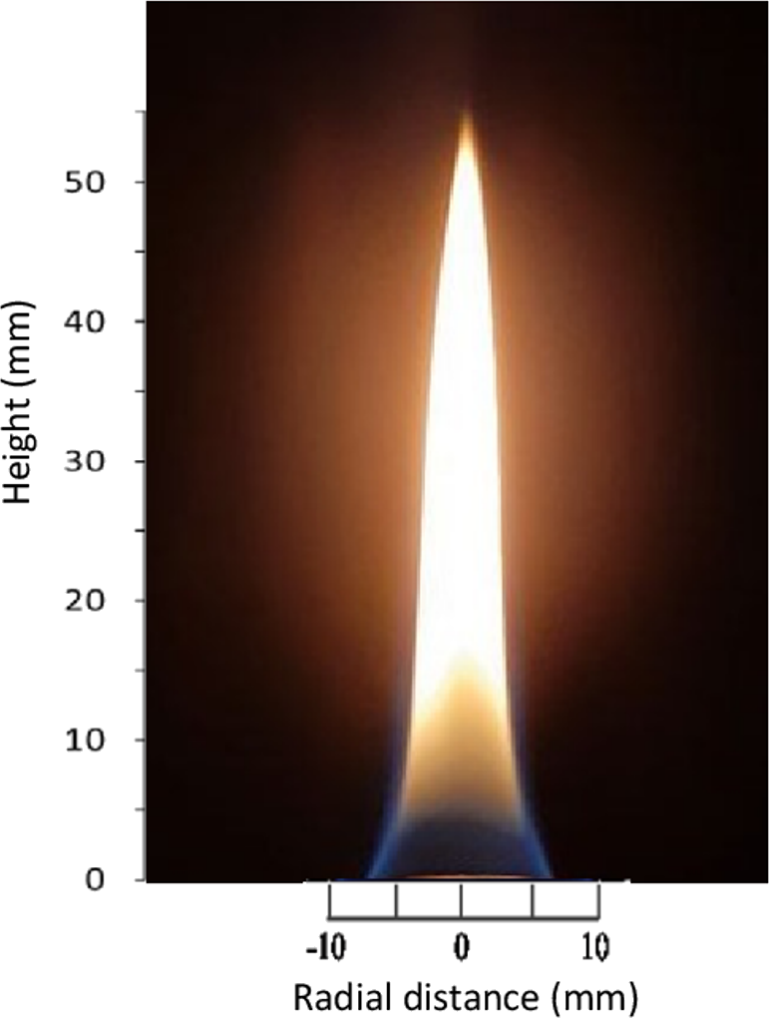
Diffusion flame of 0.05 slpm of LPG and 2 slpm of air (Φ = 0.77).

The flames were characterized regarding flame shape and temperature distribution at the center of the flame. Figure 2 shows a line-of-sight image of the stable diffusion flame, burning at lean combustion with Φ = 0.77, where the equivalence ratio was calculated based on the inlet conditions. The diffusion flame has a bright yellow color, due to soot formation, and a height of about 55 mm. It exhibits the typical blue coloration at the bottom due to the efficient combustion of the fuel and oxidizer mixture. The blue hue indicates a more complete combustion process, where the fuel is burning efficiently. The flame is stable, and burning straight with upward propagation. It also shows no soot formation on the impregnated beads when the beads are held on top of the flame.

**Figure 3:**
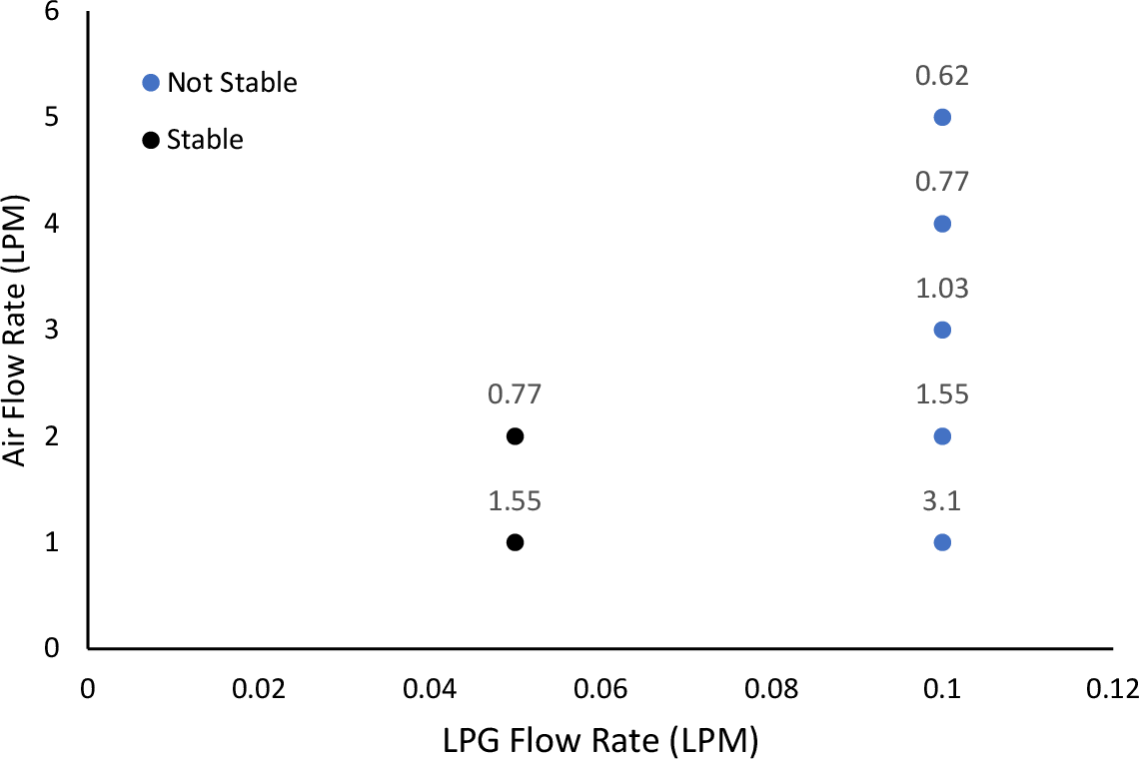
Stability of diffusion flames using LPG and air as inlet gases. The equivalence ratio is shown on the data labels.

LPG–air diffusion flames were used to study the flame stability in the ﬂammability limit for upward propagation of Φ = 0.53–2.80 [21]. Air and LPG are passed through different inlets to the burner without mixing prior to combustion. As shown in Figure 3, the flame is unstable at 0.10 slpm of LPG with continuous flickering, whereas at 0.05 slpm of LPG, the flame has a very stable shape and burns continuously. Lower flow rates allow more time for mass diffusion and mixing, resulting in a more stable flame. Figure 3 also suggests that as the flow rate of LPG increases, a higher flow rate of air is required for stability. The flame with Φ = 1.55 at 0.05 slpm of LPG shows the presence of soot when impregnated beads are employed. This indicates the competition between CNF and soot formation, which is not favorable for the growth of the former.

**Figure 4:**
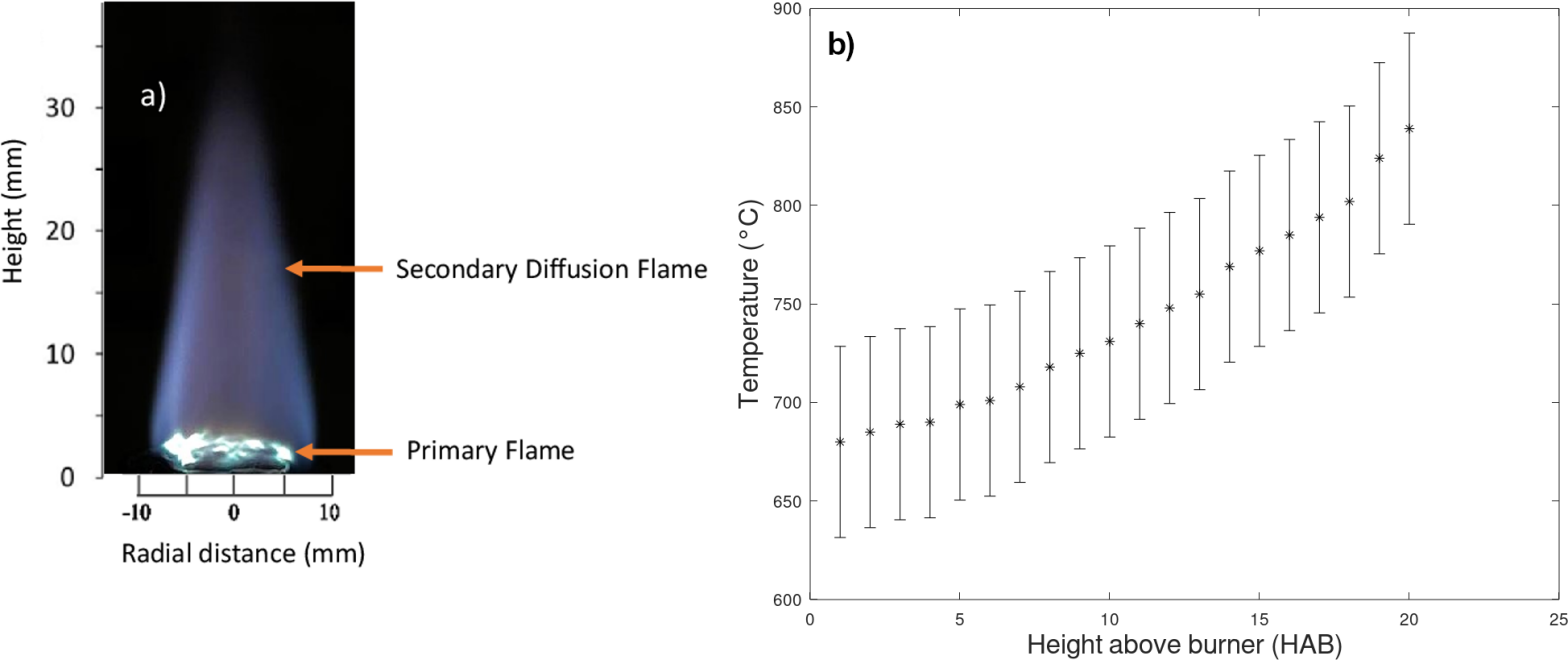
Premixed flame of LPG, nitrogen, and oxygen. (a) Flame at an equivalence ratio of 1.8. (b) The corresponding temperature at the flame centerline as function of the height above burner.

Stable flame conditions of Φ = 0.77 were used to achieve a premixed flame. Because it shows no formation of soot, this LPG premixed flame was utilized to conduct the synthesis experiments. As the flow rate of air is increased, the fuel-to-air ratio changes, which dilutes the concentration of LPG in the combustion zone, reaching the lean burning limit. The flow rate of air was divided between nitrogen and oxygen according to their weight fraction in air, and the corresponding flow rates were increased until the premixed flame was achieved as shown in Figure 4a.

Premixed flames tend to be more stable and better controlled than diffusion flames. The premixed flame was created using LPG, oxygen, and nitrogen as shown in Figure 4a. In a premixed flame setup, the use of sintered metal allows for a uniform gas flow distribution producing a flat flame shape at the top of the sintered metal. The secondary diffusion flame is formed by the entrainment of ambient air. The flame is moving upwards at an equivalence ratio of 1.8, that is, burning at rich conditions. In a rich mixture, the excess fuel in the reaction draws the surrounding air, which provides additional oxygen that mixes with the excess fuel. This process allows for continuous combustion further from the source, producing a secondary diffusion flame as shown in the image in Figure 4a. The flame height based on the tip of the secondary diffusion flame is about 25 mm. A diffusion flame forms at the stoichiometric equivalence ratio where the fuel and the oxidizer are present in ideal proportions for complete combustion, resulting the secondary diffusion flame burn at similar temperatures regardless of the equivalence ratio. The synthesis experiments were conducted with a wire mesh at 10 mm HAB supported with a funnel. During the growth process, the wire mesh quenches the secondary diffusion flame, which allows the thermal conditions of the growth to be influenced solely by the premixed flame front.

The corresponding temperature distribution graph in Figure 4b shows the temperature of the flame at the center of the burner at varying HAB values. For CNT growth, the ideal temperatures are between 750 and 950 °C [23–24]; for CNF growth, the ideal temperatures are between 550 and 650 °C [14,17]. The CNT growth regions are identified at higher HAB values and near the flame center [25]. The highest temperature is observed at 20 mm HAB, and the lowest is at 1 mm HAB. Higher nitrogen concentrations at 1 mm dilute the reactive components, spreading the combustion energy over more molecules and lowering the flame temperature. The temperature of the premixed flame using LPG behaves in a different manner than the temperature of a premixed flat flame using methane because of the existence of the secondary diffusion flame. In a premixed flat flame of methane, the temperature decreases as the height above burner increases [16], while in the LPG premixed flame, the secondary diffusion flame burns at stoichiometric equivalence ratio, increasing the overall temperature of the flame. The temperature distribution in the center of ethanol diffusion ﬂame studied by Pan et al. showed results similar to those of the LPG flame in Figure 4b [26]. The wire mesh lowers the temperature below 731 °C at 10 mm HAB. Wei et al. used temperatures around 550 °C for CNF growth [15]. As the height increases from 1 mm, where decomposition occurs around 650 °C (Figure 4b), the premixed flame temperature drops in the absence of a secondary diffusion flame.

### Nanomaterial growth and characterization

**Figure 5:**
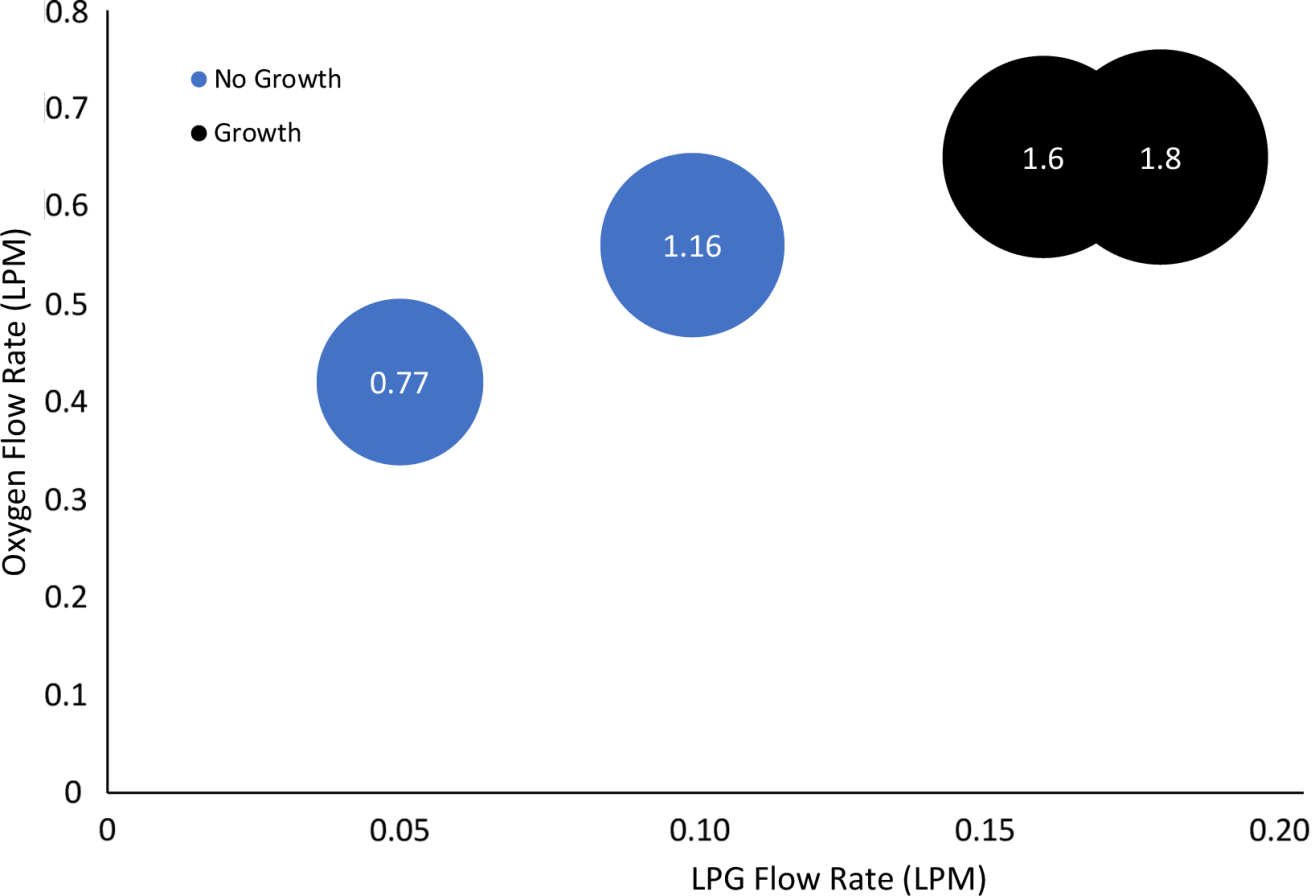
Growth regime using the premixed flame of LPG (the nitrogen flow rates determine the size of the circles, and the equivalence ratio is shown in the data labels).

The equivalence ratio values were calculated using the equation


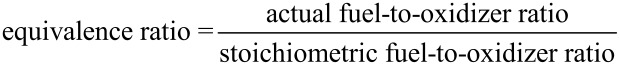


and are summarized in Table 1. Butane was considered the main component of LPG [27]. The equivalence ratio was increased to determine the growth regime for the synthesis process. The growth and no-growth regions for CNF synthesis were determined, and the results are shown in Figure 5.

**Table 1:** Variation of the flow rates and the corresponding equivalence ratios.

C_4_H_10_ + 6.5O_2_ + 25N_2_ → 4CO_2_ + 5H_2_O + 25N_2_		

Flow rate (slpm)		Equivalence ratio (Φ)
LPG	oxygen	

0.05	0.42	0.77
0.10	0.56	1.16
0.16	0.65	1.60
0.18	0.65	1.80

Zirconia beads with were placed at 10 mm HAB in a funnel to conduct the experiment. A range of flame conditions was employed to study the growth regime at varying equivalence ratios. The result showed that there was no growth on the beads at lower equivalence ratios as shown in Figure 5. At lower equivalence ratios, the mixture is lean, meaning there is more oxidizer relative to fuel, which results in a lower concentration of carbon atoms available for CNF growth. At equivalence ratios of 1.60 and 1.80, the concentration of carbon atoms is higher, which facilitates the growth of CNF, and growth is seen on the beads. The growth rate is higher at Φ = 1.8 than at Φ =1.60.

**Figure 6:**
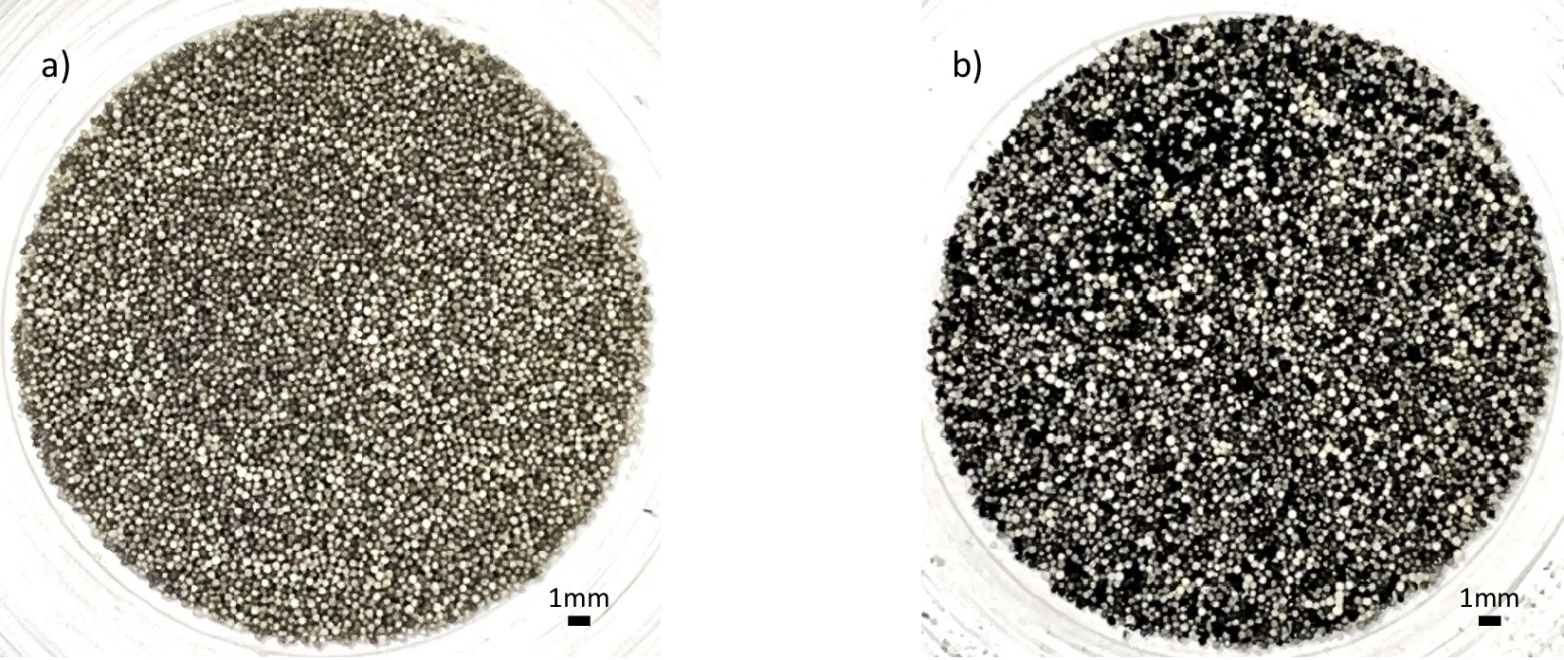
Zirconia beads (a) before synthesis (b) after synthesis. Scale bars: 1 mm.

Figure 6 shows zirconia beads before and after synthesis. Before synthesis, the impregnated zirconia beads were gray. They turned black after the synthesis because of a layer of CNF deposited on the surface of the beads. As the equivalence ratio increased, a visible black layer formed on the beads, as shown in Figure 6b. The deposited CNFs were extracted from the beads using ex situ collection with a fluidized bed, and the black CNF powder was used for further characterization.

**Figure 7:**
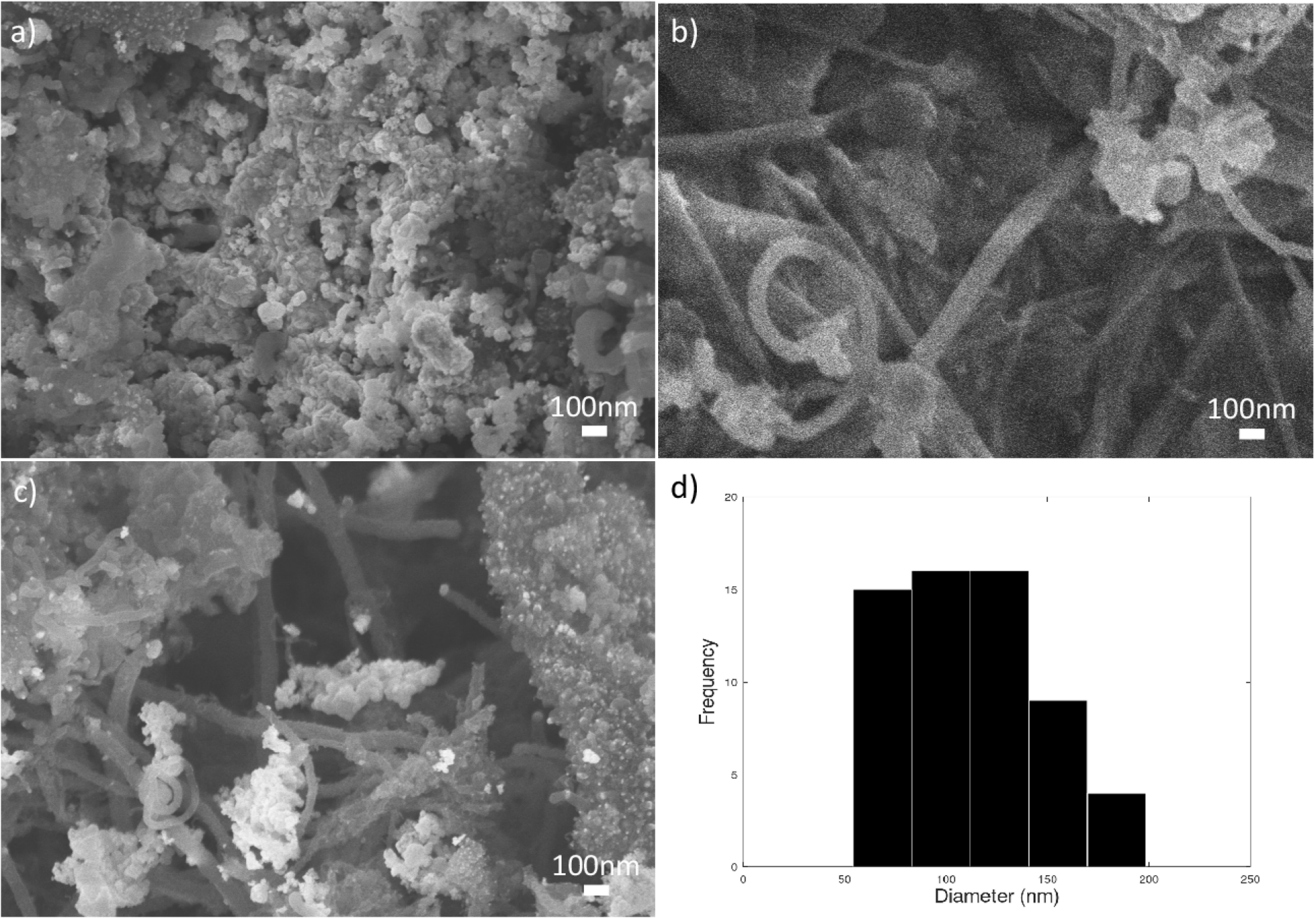
FESEM images of CNFs (Φ = 1.60). (a) Amorphous carbon. (b) CNFs of ring-like shape. (c) Randomly oriented CNFs with amorphous carbon. (d) Diameter distribution of CNFs found in the sample.

The FESEM images in Figure 7 show the CNFs grown on the substrate. The CNFs show ring-like structures (Figure 7b) and randomly oriented long shapes (Figure 7c). A large amount of amorphous carbon is shown in Figure 7a. The amorphous carbon could be due to the presence of the catalyst that did not grow and is left in the sample [28]. The sample seems to have more amorphous carbon than CNFs, and the diameter of the deposited CNFs is larger than that of CNTs.

The histogram in Figure 7d shows the distribution of the diameter from the FESEM images. The diameter of CNFs is larger than that of normal CNTs. FESEM images revealed the lowest CNF diameter at approximately 54 nm, which is larger than the common CNT dimensions (≈12 nm). The CNF diameters have a mean value of about 114 nm, while most of the nanofiber diameters range from 60 to 100 nm [14]. The histogram is skewed to the left, meaning there are more samples with smaller diameter of CNF and fewer samples with higher values. This suggests that most samples have a relatively low number of CNFs grown. Slight variations in temperature with higher equivalence ratio can affect the number of CNFs grown.

**Figure 8:**
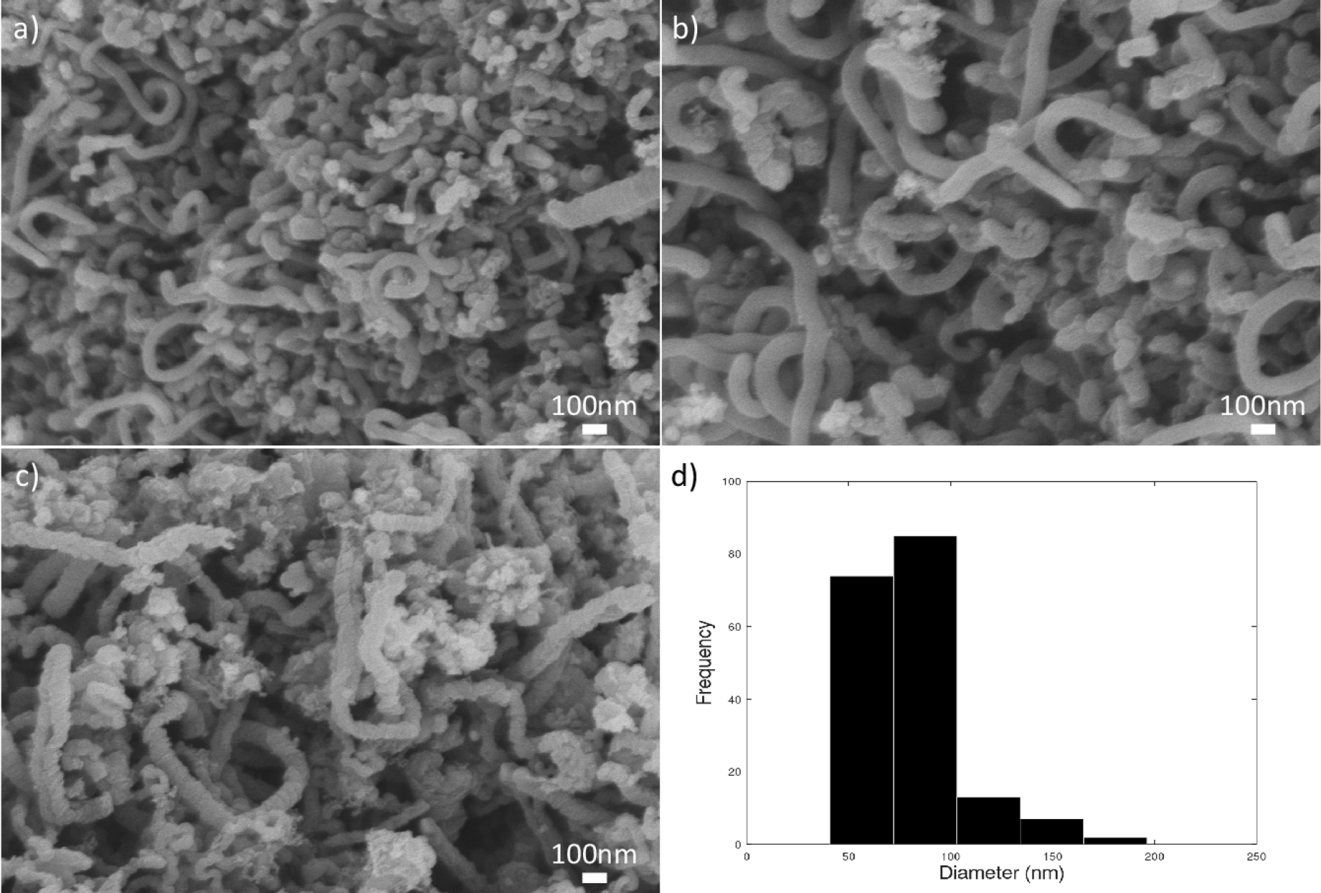
FESEM images of CNF (Φ = 1.80). (a–c) Randomly oriented CNFs. (d) Diameter distribution of CNFs found in the sample.

Another experiment was conducted under the same synthesis condition with an equivalence ratio of 1.80. As expected, there is large number of CNFs present in the sample, as shown in Figure 8. The fraction of amorphous carbon in this sample is much smaller than that in Figure 7. The flame at 1.80 equivalence ratio ensures a suitable environment for carbon deposition. At higher fuel concentrations, the amount of available carbon is higher, which provides more carbon atoms that can interact with the catalyst’s surface, enhancing the ability of the catalyst to decompose the carbon source to grow CNF structures.

The histogram in Figure 8b shows the diameter distribution of CNFs in a more ordered growth. Most of the CNFs have a diameter below 100 nm, and the variation is less scattered. The average diameter of CNFs in the sample in Figure 8 is around 78 nm, which is significantly lower than the average diameter of the sample in Figure 7. The highest frequency of diameters observed is around 72 to 103 nm. The smallest was around 20 nm, while the largest was around 200 nm (Figure 6d and Figure 7d). As found in the study of Pan et al. [26], average diameters of CNFs are below 100 nm, and it is evident that the nanomaterial found in this sample is CNFs because of the entangled structure and the larger diameters compared to CNTs.

As the equivalence ratio decreases from rich condition towards stoichiometry conditions, the flame temperature starts to decrease as the flame is leaner than the stoichiometry condition. The increase in particle size is a result of the increased mobility and leads to a higher likelihood of particles sticking together [29]. This phenomenon explains the production of CNFs with large diameters. As the equivalence ratio reduces from Φ = 1.80 to Φ = 1.00, the primary flame temperature increases. At higher temperatures, the catalyst particle size increases, which increases the mobility of the particle and the chances to collide and accumulate, results larger diameter of CNF.

An equivalence ratio of 1.60 favors the formation of amorphous carbon, which can be attributed also to the reaction conditions. The thermal conditions at an equivalence ratio of 1.60 lead to a higher-temperature environment, which accelerates the breakdown of hydrocarbon molecules into free carbon atoms. The carbon atoms, in the absence of efficient catalytic activity, aggregate to form amorphous carbon rather than ordered structures such as CNFs as shown in Figure 7a. In contrast, at an equivalence ratio of 1.80, despite a higher carbon supply rate, the lower temperature does not sufficiently drive the decomposition reaction, thus providing a more conducive environment for the growth of ordered carbon structures as shown in Figure 8a [30].

**Figure 9:**
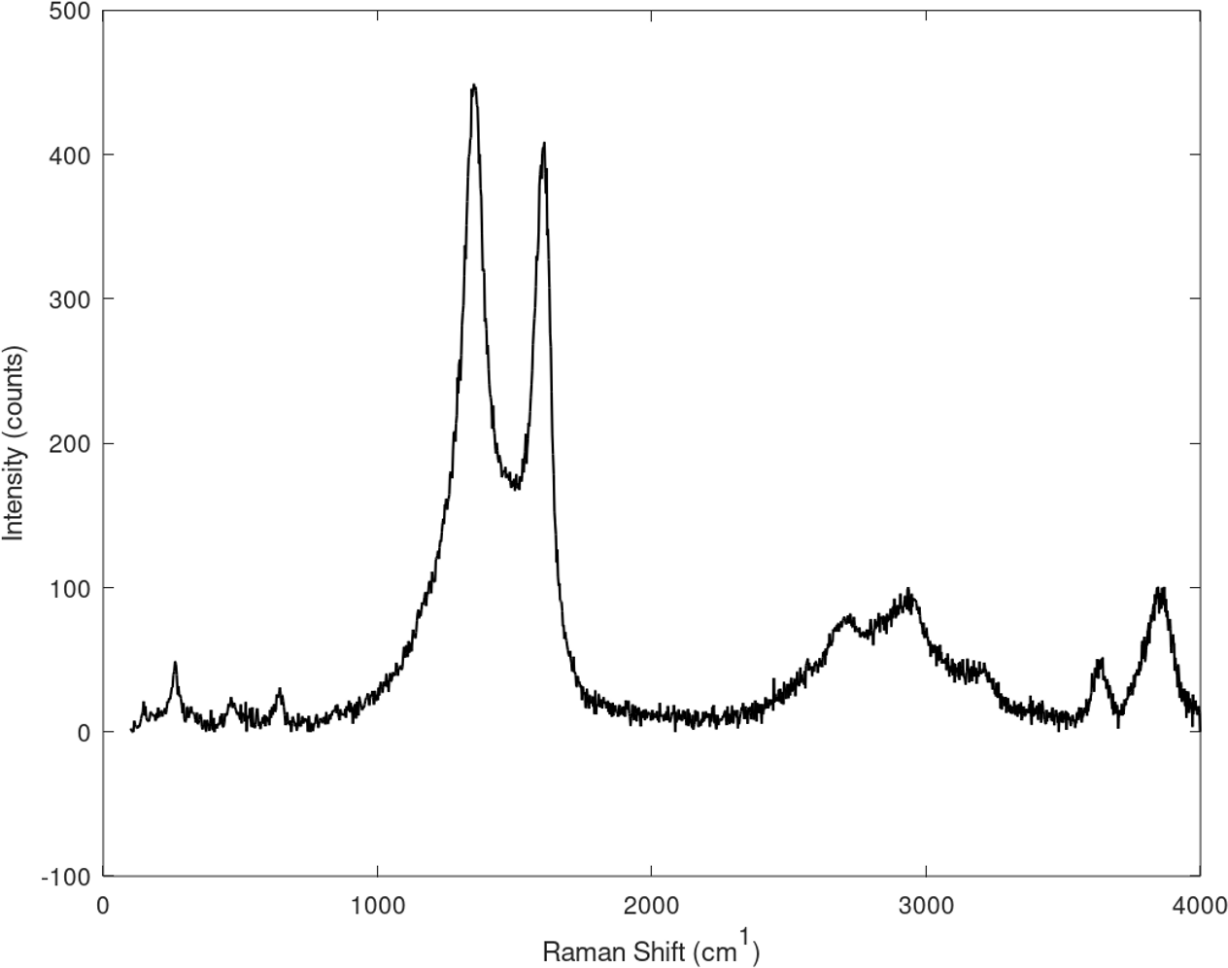
Raman shifts of CNFs (Φ = 1.80).

The Raman spectra in Figure 9 show the *I*_D_ and *I*_G_ peaks at around 1300–1400 cm^−1^ and 1500–1600 cm^−1^, respectively, for the CNFs synthesized Φ = 1.8. These peaks prove that CNFs are present in the sample. The peak of the D band is larger than that of the G band, which means that the CNFs in the sample have more defects or disorders than crystalline CNFs. A higher presence of defects was found before in CNFs via Raman spectroscopy [12]. A slightly elevated defect density was also observed in CNTs derived from LPG [16,31]. The D band and G band intensities show an *I*_D_/*I*_G_ ratio of more than 1.13, which is a clear indicator for high disorder and numerous defects in the CNFs [32].

## Conclusion

The synthesis of CNFs using a premixed flame with LPG remains a challenging topic, but it is a useful study for the economical production of CNFs at large scale. Because of the limited number of available studies regarding the use of LPG premixed flames for CNF synthesis, the growth regimes with a range of equivalence ratios was observed. The present study explores the CNF growth on nickel-impregnated zirconia beads using the flame synthesis method with a premixed flame of LPG. The analysis of the temperature distribution of the LPG premixed flame helps to understand the deposition temperature, which can provide sufficient energy for the nucleation to start. A LPG premixed flame with secondary diffusion flame is stable in the equivalence ratio range of 0.77 to 1.80, burning continuously with no flicker. The premixed flame front provides maximum growth temperatures below 650 °C, while the diffusion flame front may provide more than 700 °C of maximum growth temperature at an HAB of 10 mm. The experiment shows that there is no growth on the substrate at equivalence ratios of 0.77 and 1.16, but growth can be seen at equivalence ratios of 1.60 and 1.80. The diameter of the CNFs increases as the equivalence ratio decreases, which eventually leads to the formation of amorphous carbon as observed at an equivalence ratio of 1.60. At an equivalence ratio of 1.6 in a high-temperature environment, the hydrocarbons dissociate to carbon atoms very rapidly. These free carbon atoms attach on the catalyst particle. Because of the inefficiency of the catalyst, the excess carbon atoms keep attaching and eventually forming amorphous carbons. The higher temperature also leads to the increased catalyst particle size, results in a higher chance of collision and accumulation, hence the larger diameter of CNFs at an equivalence ratio of 1.60 compared to the equivalence ratio of 1.80.

## Data Availability

All data that supports the findings of this study is available in the published article and/or the supporting information of this article.
